# Supplementary material for: Mixed-Methods Approach: Impact of Clinical Consenter Diversity on Clinical Trials Enrollment
Source: Cancers (Basel). 2025 Mar 20;17(6):1043. doi: 10.3390/cancers17061043 (PMC11941056; doi:10.3390/cancers17061043)
Supplement: Supplementary file 1 [file cancers-17-01043-s001.zip › cancers-3490762-supplementary.docx]

**Table S1.** Survey Questionnaire Questions and References.

|  | **Questions/Variables** | **Reference** | **Original Reference** | **Changes made for our survey/Notes** | |
| --- | --- | --- | --- | --- | --- |
| Q1 | 1. Before you agreed to participate in this study, you were invited to participate in a different clinical research study that involved the collection of leftover tissue and blood. Please indicate how much you agree with the following statements regarding the communication with the person that previously spoke to you: |  |  |  | |
| v1 | I understood that entry into the clinical research study was voluntary | Jenkins et al., 2013 [8]. Drivers and barriers to patient participation in RCTs. *British journal of cancer*, *108*(7), 1402. | Jenkins et al., 2005 [9]. Discussing randomized clinical trials of cancer therapy: evaluation of a Cancer Research UK training programme. *bmj*, *330*(7488), 400. | Adapted from Jenkins 2013 - Changed "trial" to "clinical research study"; no mention of validation in either article | |
| v2 | I understood that if I agreed to join the clinical research study, I could leave at any time | Jenkins et al., 2013 [8]. Drivers and barriers to patient participation in RCTs. *British journal of cancer*, *108*(7), 1402. | Jenkins et al., 2005 [9]. Discussing randomized clinical trials of cancer therapy: evaluation of a Cancer Research UK training programme. *bmj*, *330*(7488), 400. | Adapted from Jenkins 2013 -Changed "trial" to "clinical research study"; no mention of validation in either article | |
| v3 | I felt that the consenter was sensitive to my concerns | Jenkins et al., 2013 [8]. Drivers and barriers to patient participation in RCTs. *British journal of cancer*, *108*(7), 1402. | Jenkins et al., 2005 [9]. Discussing randomized clinical trials of cancer therapy: evaluation of a Cancer Research UK training programme. *bmj*, *330*(7488), 400. | Adapted from Jenkins 2013 -Changed "health care professional" to "consenter"; no mention of validation in either article | |
| v4 | I was given the opportunity to ask questions | Jenkins et al., 2013 [8]. Drivers and barriers to patient participation in RCTs. *British journal of cancer*, *108*(7), 1402. | Jenkins et al., 2005 [9]. Discussing randomized clinical trials of cancer therapy: evaluation of a Cancer Research UK training programme. *bmj*, *330*(7488), 400. | | Adapted from Jenkins 2013 -no change, same wording; no mention of validation in either article |
| v5 | I felt the consenter listened to what I have to say | Jenkins et al., 2013 [8]. Drivers and barriers to patient participation in RCTs. *British journal of cancer*, *108*(7), 1402. | Jenkins et al., 2005 [9]. Discussing randomized clinical trials of cancer therapy: evaluation of a Cancer Research UK training programme. *bmj*, *330*(7488), 400. | | Adapted from Jenkins 2013-Changed "HCP" to "consenter"; no mention of validation in either article |
| v6 | I felt that the consenter gave me all the information I needed to make a decision | Jenkins et al., 2013 [8]. Drivers and barriers to patient participation in RCTs. *British journal of cancer*, *108*(7), 1402. | Jenkins et al., 2005 [9]. Discussing randomized clinical trials of cancer therapy: evaluation of a Cancer Research UK training programme. *bmj*, *330*(7488), 400. | | Adapted from Jenkins 2013- Changed "HCP" to "consenter"; no mention of validation in either article |
| v7 | I felt that the consenter created an atmosphere of trust and support | Jenkins et al., 2013 [8]. Drivers and barriers to patient participation in RCTs. *British journal of cancer*, *108*(7), 1402. | Jenkins et al., 2005 [9]. Discussing randomized clinical trials of cancer therapy: evaluation of a Cancer Research UK training programme. *bmj*, *330*(7488), 400. | | Adapted from Jenkins 2013 - Changed "HCP" to "consenter"; no mention of validation in either article |
| v8 | I felt that the consenter was empathetic to my situation | Heiney et al., 2010 [11]. Evaluation of conceptual framework for recruitment of African American patients with breast cancer. In *Oncology nursing forum* (Vol. 37, No. 3, p. E160). NIH Public Access | Overholser. 2007 [10]. The central role of the therapeutic alliance: A simulated interview with Carl Rogers. *Journal of Contemporary Psychotherapy*, *37*(2), 71-78. | | Idea from Heiney; not a validated measure |
| v9 | I felt empowered by the consenter to make my own decision regarding my participation | Heiney et al., 2010 [11]. Evaluation of conceptual framework for recruitment of African American patients with breast cancer. In *Oncology nursing forum* (Vol. 37, No. 3, p. E160). NIH Public Access | Overholser. 2007 [10].  The central role of the therapeutic alliance: A simulated interview with Carl Rogers. *Journal of Contemporary Psychotherapy*, *37*(2), 71-78. | | Idea from Heiney; not a validated measure |
| Q2, v10 | Did you agree to participate in the previous study (regarding leftover tissue and/or blood)? | Seewaldt lab |  | |  |
| Q3, v11 | How many times have you been previously invited to participate in a clinical research study (not including this one)? | Seewaldt lab |  | |  |
| Q4, v12 | How many clinical research studies have you participated in (not including this one)? | Seewaldt lab |  | |  |
| Q5, v13 | Do you know someone who has participated in a clinical research study? | Seewaldt lab |  | |  |
| v14 | If yes, what is their relationship to you? Family, Friend, Spouse, co-worker, other: _____ | Seewaldt lab |  | |  |
| Q6 | How important are the following characteristics in someone who asks you to participate in any clinical research study? |  |  | |  |
| v15 | The person asking me is of my racial/ethnic group | Myles et al., 2018 [15].  A Multicenter Investigation of Factors Influencing Women's Participation in Clinical Trials. *Journal of Women's Health*, *27*(3), 258-270. | Kurt et al., 2017 [12].  Racial differences among factors associated with participation in clinical research trials. *Journal of racial and ethnic health disparities*, *4*(5), 827-836. | | Adapted from Myles, they used "the doctor conducting the research is the same race/ethnicity as me" |
| v16 | They look like people in my community | Seewaldt lab |  | |  |
| v17 | They speak my language | Myles et al., 2018 [15].  A Multicenter Investigation of Factors Influencing Women's Participation in Clinical Trials. *Journal of Women's Health*, *27*(3), 258-270. | Kurt et al., 2017 [12].  Racial differences among factors associated with participation in clinical research trials. *Journal of racial and ethnic health disparities*, *4*(5), 827-836. | | Adapted from Myles, they used "the doctor conducting the research speaks the same language as I do" |
| v18 | They are the same gender as me | Myles et al., 2018 [15].  A Multicenter Investigation of Factors Influencing Women's Participation in Clinical Trials. *Journal of Women's Health*, *27*(3), 258-270. | Kurt et al., 2017 [12].  Racial differences among factors associated with participation in clinical research trials. *Journal of racial and ethnic health disparities*, *4*(5), 827-836. | | Adapted from Myles; they used "the doctor conducting the research is the same gender (sex) as me" |
| v19 | They are willing to be more flexible with my schedule | Heller et al., 2014 [16]. Strategies addressing barriers to clinical trial enrollment of underrepresented populations: a systematic review. *Contemporary clinical trials*, *39*(2), 169-182. | n/a | | Idea from Heller (lit review study) |
| v20 | They are empathetic to my situation | Heiney et al., 2010 [11]. Evaluation of conceptual framework for recruitment of African American patients with breast cancer. In *Oncology nursing forum* (Vol. 37, No. 3, p. E160). NIH Public Access | Overholser. 2007 [10]. The central role of the therapeutic alliance: A simulated interview with Carl Rogers. *Journal of Contemporary Psychotherapy*, *37*(2), 71-78. | | Idea from Heiney - not a validated measure |
| v21 | They take the time to listen to my concerns | Heiney et al., 2010 [11]. Evaluation of conceptual framework for recruitment of African American patients with breast cancer. In *Oncology nursing forum* (Vol. 37, No. 3, p. E160). NIH Public Access | Overholser. 2007 [10].  The central role of the therapeutic alliance: A simulated interview with Carl Rogers. *Journal of Contemporary Psychotherapy*, *37*(2), 71-78. | | Idea from Heiney - not a validated measure |
| v22 | They make me feel empowered to make my own decision regarding my participation in the clinical research study | Heiney et al., 2010 [11]. Evaluation of conceptual framework for recruitment of African American patients with breast cancer. In *Oncology nursing forum* (Vol. 37, No. 3, p. E160). NIH Public Access | Overholser. 2007 [10].  The central role of the therapeutic alliance: A simulated interview with Carl Rogers. *Journal of Contemporary Psychotherapy*, *37*(2), 71-78. | | Idea from Heiney - not a validated measure |
| Q7 | Please indicate how important the following factors are in your decision to participate in any clinical research study. |  |  | |  |
| v23 | Knowledge learned from my participation will benefit someone in the future | Myles et al., 2018 [15].  A Multicenter Investigation of Factors Influencing Women's Participation in Clinical Trials. *Journal of Women's Health*, *27*(3), 258-270. | Kurt et al., 2017 [12].  Racial differences among factors associated with participation in clinical research trials. *Journal of racial and ethnic health disparities*, *4*(5), 827-836. | | Adapted from Myles - same wording (see page 4 of Myles for potential validation) |
| v24 | To benefit future generations of my own family | Locock, & Smith, 2011 [13]. Personal benefit, or benefiting others? Deciding whether to take part in clinical trials. *Clinical trials*, *8*(1), 85-93. | n/a | | Idea from Locock (qualitative interview study) |
| v25 | To acknowledge contributions made by past generations to research | Locock, & Smith, 2011 [13]. Personal benefit, or benefiting others? Deciding whether to take part in clinical trials. *Clinical trials*, *8*(1), 85-93. | n/a | | Idea from Locock (qualitative interview study) |
| v26 | My desire to please my doctor | Myles et al., 2018 [15].  A Multicenter Investigation of Factors Influencing Women's Participation in Clinical Trials. *Journal of Women's Health*, *27*(3), 258-270. | Kurt et al., 2017 [12].  Racial differences among factors associated with participation in clinical research trials. *Journal of racial and ethnic health disparities*, *4*(5), 827-836. | | Adapted from Myles - same wording (see page 4 of Myles for potential validation) |
| v27 | Getting paid to participate | Cortés et al., 2017 [17]. Urban-Dwelling Community Members’ Views on Biomedical Research Engagement. *Qualitative health research*, *27*(1), 130-137. | n/a | | Idea from Cortes (qualitative study, focus groups) |
| v28 | My religious beliefs | Myles et al., 2018 [15].  A Multicenter Investigation of Factors Influencing Women's Participation in Clinical Trials. *Journal of Women's Health*, *27*(3), 258-270. | Kurt et al., 2017 [12].  Racial differences among factors associated with participation in clinical research trials. *Journal of racial and ethnic health disparities*, *4*(5), 827-836. | | Adapted from Myles - same wording (see page 4 of Myles for potential validation) |
| v29 | My trust in research | Cortés et al., 2017 [17]. Urban-Dwelling Community Members’ Views on Biomedical Research Engagement. *Qualitative health research*, *27*(1), 130-137. | n/a | | Idea from Cortes (qualitative study, focus groups) |
| v30 | How much time it will require to participate | Myles et al., 2018 [15].  A Multicenter Investigation of Factors Influencing Women's Participation in Clinical Trials. *Journal of Women's Health*, *27*(3), 258-270. | Kurt et al., 2017 [12].  Racial differences among factors associated with participation in clinical research trials. *Journal of racial and ethnic health disparities*, *4*(5), 827-836. | | Adapted from Myles- they have "time commitment" (see page 4 of Myles for potential validation) |
| v31 | Risk of unknown side effects or other risks | Myles et al., 2018 [15].  A Multicenter Investigation of Factors Influencing Women's Participation in Clinical Trials. *Journal of Women's Health*, *27*(3), 258-270. | Kurt et al., 2017 [12].  Racial differences among factors associated with participation in clinical research trials. *Journal of racial and ethnic health disparities*, *4*(5), 827-836. | | Adapted from Myles - same wording (see page 4 of Myles for potential validation) |
| v32 | Having support from a family member, spouse, or friend to participate | Myles et al., 2018 [15.  ] A Multicenter Investigation of Factors Influencing Women's Participation in Clinical Trials. *Journal of Women's Health*, *27*(3), 258-270. | Kurt et al., 2017 [12].  Racial differences among factors associated with participation in clinical research trials. *Journal of racial and ethnic health disparities*, *4*(5), 827-836. | | Adapted from Myles - they have "my family's concern" (see page 4 of Myles for potential validation) |
| v33 | Feeling a sense of satisfaction in 'doing the right thing' | Locock, & Smith, 2011 [13]. Personal benefit, or benefiting others? Deciding whether to take part in clinical trials. *Clinical trials*, *8*(1), 85-93. | Bulmer,1986 [14]. *Neighbours: the work of Philip Abrams*. Cambridge University Press. | | Idea from Locock (qualitative interview study) |
| v34 | Feeling too overwhelmed with my medical history and/or current diagnosis | Seewaldt lab |  | | Patients who declined would tell us that they were feeling overwhelmed at that moment |
